# Supplementary material for: Efficacy and Safety of Polyunsaturated Fatty Acids Supplementation in the Treatment of Attention Deficit Hyperactivity Disorder (ADHD) in Children and Adolescents: A Systematic Review and Meta-Analysis of Clinical Trials
Source: Nutrients. 2021 Apr 8;13(4):1226. doi: 10.3390/nu13041226 (PMC8068201; doi:10.3390/nu13041226)
Supplement: Supplementary file 1 [file nutrients-13-01226-s001.zip › Table S2_Search protocol.docx]

**Search protocol**

| Project title | Efficacy and safety of polyunsaturated fatty acids supplemen-tation in the treatment of Attention Deficit Hyperactivity Dis-order (ADHD) in children and adolescents: a systematic review and meta-analysis of clinical trials |
| --- | --- |
| Search specialist | Kirsten Birkefoss |
| Latest update | 19.06.2020 |

**Information souces**

| **DATABASES** | **INTERFACE** | **DATE OF SEARCH** |
| --- | --- | --- |
| **Medline** | OVID | 19.06.2020 |
| **Embase** | OVID | 19.06.2020 |
| **PsycInfo** | OVID | 19.06.2020 |
| **Cinahl** | EBSCo | 19.06.2020 |

**Medline**

Database(s): **Ovid MEDLINE(R) and Epub Ahead of Print, In-Process & Other Non-Indexed Citations, Daily and Versions(R)**1946 to June 18, 2020
Search Strategy:

| **#** | **Searches** | **Results** |
| --- | --- | --- |
| 1 | exp "Fatty acids, unsaturated"/ | 225146 |
| 2 | exp Diet therapy/ | 54206 |
| 3 | exp Fish oils/ | 27606 |
| 4 | exp Carnitine/ | 9667 |
| 5 | ((fatty adj1 acid*) or ((Polyunsaturated or poly-unsaturated or unsaturated) adj1 (fat or fatty)) or omega-3 or omega3 or omega 3 or omega-6 or omega6 or omega 6 or (docosahexaenoic adj acid*) or (eicosapentaenoic adj acid*) or (arachidonic adj acid)).ti,bt,ab,kf. | 251366 |
| 6 | ((fish adj1 oil*) or cod liver oil* or lax oil* or tuna oil* or carnitine or Levocarnitine or "L Carnitine" or L-carnitine or bicarnitine).ti,bt,ab,kf. | 26267 |
| 7 | ((diet* or nutrition or food) adj1 (therapy or supplement*)).ti,bt,ab,kf. | 45777 |
| 8 | or/1-7 | 508829 |
| 9 | exp Attention Deficit Disorder with Hyperactivity/ | 28517 |
| 10 | (ADHD or ADDH or (hyperkinetic adj1 disorder*) or (Attention adj1 Deficit adj1 Disorder) or (attention-deficit adj1 disorder)).ti,bt,ab,kf. | 27285 |
| 11 | 9 or 10 | 36770 |
| 12 | 8 and 11 | 392 |
| 13 | limit 12 to (randomized controlled trial or controlled clinical trial) | 62 |
| 14 | (((random* or cluster-random* or quasi-random* or control?ed or crossover or cross-over or blind* or mask*) adj4 (trial*1 or study or studies or analy*)) or rct).ti,bt,ab,kf,hw. | 1057864 |
| 15 | (placebo* or single-blind* or double-blind* or triple-blind*).ti,bt,kf,hw. | 224515 |
| 16 | ((single or double or triple) adj2 (blind* or mask*)).ti,bt,kf,hw. | 195882 |
| 17 | ((patient* or person* or participant* or population* or allocat* or assign*) adj3 random*).ti,bt,ab,kf. | 246898 |
| 18 | 14 or 15 or 16 or 17 | 1141746 |
| 19 | 12 and 18 | 148 |
| 20 | 13 or 19 | 148 |
| 21 | limit 20 to (yr="2017-2020" and (english or danish or german or norwegian or swedish)) | 29 |

**Embase**

Database(s): **Embase**1974 to 2020 June 18
Search Strategy:

| **#** | **Searches** | **Results** |
| --- | --- | --- |
| 1 | exp unsaturated fatty acid/ | 150025 |
| 2 | Diet therapy/ or diet supplementation/ | 135720 |
| 3 | exp Fish oil/ | 16824 |
| 4 | exp Carnitine/ | 15507 |
| 5 | ((fatty adj1 acid*) or ((Polyunsaturated or poly-unsaturated or unsaturated) adj1 (fat or fatty)) or omega-3 or omega3 or omega 3 or omega-6 or omega6 or omega 6 or (docosahexaenoic adj acid*) or (eicosapentaenoic adj acid*) or (arachidonic adj acid)).ti,ab,kw. | 293087 |
| 6 | ((fish adj1 oil*) or cod liver oil* or lax oil* or tuna oil* or carnitine or Levocarnitine or "L Carnitine" or L-carnitine or bicarnitine).ti,ab,kw. | 33664 |
| 7 | ((diet* or food or nutrition) adj1 (therapy or supplement*)).ti,ab,kw. | 56194 |
| 8 | or/1-7 | 508241 |
| 9 | exp Attention Deficit Disorder/ | 60740 |
| 10 | (ADHD or (hyperkinetic adj1 disorder*) or (Attention adj1 Deficit adj1 Disorder) or (attention-deficit adj1 disorder)).ti,ab,kw. | 39457 |
| 11 | 9 or 10 | 64918 |
| 12 | 8 and 11 | 1169 |
| 13 | limit 12 to (randomized controlled trial or controlled clinical trial) | 131 |
| 14 | (((random* or cluster-random* or quasi-random* or control?ed or crossover or cross-over or blind* or mask*) adj4 (trial*1 or study or studies or analy*)) or rct).ti,ab,kw. | 971123 |
| 15 | (placebo* or single-blind* or double-blind* or triple-blind*).ti,ab,kw. | 399309 |
| 16 | ((single or double or triple) adj2 (blind* or mask*)).ti,ab,kw. | 240200 |
| 17 | ((patient* or person* or participant* or population* or allocat* or assign*) adj3 random*).ti,ab,kw. | 342695 |
| 18 | 14 or 15 or 16 or 17 | 1239993 |
| 19 | 12 and 18 | 258 |
| 20 | 13 or 19 | 287 |
| 21 | limit 20 to (yr="2017-2020" and (english or danish or german or norwegian or swedish)) | 70 |

**PsycInfo**

Database(s): **APA PsycInfo**1806 to June Week 2 2020
Search Strategy:

| **#** | **Searches** | **Results** |
| --- | --- | --- |
| 1 | exp Fatty acids/ | 4971 |
| 2 | Diets/ | 12807 |
| 3 | ((fatty adj1 acid*) or ((Polyunsaturated or poly-unsaturated or unsaturated) adj1 (fat or fatty)) or omega-3 or omega3 or omega 3 or omega-6 or omega6 or omega 6 or (docosahexaenoic adj acid*) or (eicosapentaenoic adj acid*) or (arachidonic adj acid)).ti,ab,id. | 5360 |
| 4 | ((fish adj1 oil*) or cod liver oil* or lax oil* or tuna oil* or carnitine or Levocarnitine or "L Carnitine" or L-carnitine or bicarnitine).ti,ab,id. | 736 |
| 5 | ((diet* or food or nutrition) adj1 (therapy or supplement*)).ti,ab,id. | 2495 |
| 6 | 1 or 2 or 3 or 4 or 5 | 22145 |
| 7 | exp Attention Deficit Disorder/ | 27063 |
| 8 | (ADHD or (hyperkinetic adj1 disorder*) or (Attention adj1 Deficit adj1 Disorder) or (attention-deficit adj1 disorder)).ti,ab,id. | 30307 |
| 9 | 7 or 8 | 33082 |
| 10 | 6 and 9 | 235 |
| 11 | (((random* or cluster-random* or quasi-random* or control?ed or crossover or cross-over or blind* or mask*) adj4 (trial*1 or study or studies or analy*)) or rct).ti,ab,id. | 105362 |
| 12 | (placebo* or single-blind* or double-blind* or triple-blind*).ti,ab,id. | 48906 |
| 13 | ((single or double or triple) adj2 (blind* or mask*)).ti,ab,id. | 26221 |
| 14 | ((patient* or person* or participant* or population* or allocat* or assign*) adj3 random*).ti,ab,id. | 55904 |
| 15 | 11 or 12 or 13 or 14 | 162362 |
| 16 | 10 and 15 | 79 |
| 17 | limit 16 to (yr="2017-2020" and (english or danish or german or norwegian or swedish)) | 13 |

**Cinahl**

| **#** | **Query** | **Limiters/Expanders** | **Results** |
| --- | --- | --- | --- |
| S18 | S12 AND S17 | Limiters - Published Date: 20170801-20200631; Language: Danish, English, German, Norwegian, Swedish | 13 |
| S17 | S13 OR S14 OR S15 OR S16 |  | 557,993 |
| S16 | ((patient* or person* or participant* or population* or allocat* or assign*) N3 random*) |  | 334,750 |
| S15 | (placebo* or single-blind* or double-blind* or triple-blind* or ((single or double or triple) N1 (blind* or mask*)) |  | 116,255 |
| S14 | (((random* or cluster-random* or quasi-random* or control#ed or crossover or cross-over or blind* or mask*) N4 (trial* or study or studies or analy*)) or rct) |  | 535,466 |
| S13 | PT Randomized Controlled Trial |  | 131,664 |
| S12 | S8 AND S11 |  | 1,013 |
| S11 | S9 OR S10 |  | 21,922 |
| S10 | (ADHD or (hyperkinetic N1 disorder*) or (Attention N1 Deficit N1 Disorder) or (attention-deficit N1 disorder)) |  | 21,922 |
| S9 | (MH "Attention Deficit Hyperactivity Disorder") |  | 17,671 |
| S8 | S1 OR S2 OR S3 OR S4 OR S5 OR S6 OR S7 |  | 198,500 |
| S7 | ((diet* or nutrition or food) N1 (therapy or supplement*)) |  | 140,395 |
| S6 | ((fish N1 oil*) or cod liver oil* or lax oil* or tuna oil* or carnitine or Levocarnitine or "L Carnitine" or L-carnitine or bicarnitine) |  | 7,284 |
| S5 | ((fatty N1 acid*) or ((Polyunsaturated or poly-unsaturated or unsaturated) N1 (fat or fatty)) or omega-3 or omega3 or omega 3 or omega-6 or omega6 or omega 6 or (docosahexaenoic N1 acid*) or (eicosapentaenoic N1 acid*) or (arachidonic N1 acid)) |  | 36,825 |
| S4 | (MH Carnitine) |  | 1,476 |
| S3 | (MH "fish oils+") |  | 13,557 |
| S2 | (MH "Diet Therapy+") |  | 33,330 |
| S1 | (MH "Fatty Acids, Unsaturated+") |  | 28,633 |
