# Supplementary material for: Efficacy and Safety of Polyunsaturated Fatty Acids Supplementation in the Treatment of Attention Deficit Hyperactivity Disorder (ADHD) in Children and Adolescents: A Systematic Review and Meta-Analysis of Clinical Trials
Source: Nutrients. 2021 Apr 8;13(4):1226. doi: 10.3390/nu13041226 (PMC8068201; doi:10.3390/nu13041226)
Supplement: Supplementary file 1 [file nutrients-13-01226-s001.zip › Table S3_ADHD PUFA_Excluded studies.pdf]

# Characteristics of studies

## Characteristics of excluded studies

### *Abdullah 2019*

| | |
|---|---|
| **Reason for exclusion** | Wrong study design |

### *Anand 2016*

| | |
|---|---|
| **Reason for exclusion** | Wrong study design |

### *Anjos 2013*

| | |
|---|---|
| **Reason for exclusion** | Wrong patient population |

### *Arnold 2013*

| | |
|---|---|
| **Reason for exclusion** | Wrong study design |

### *Assareh 2017*

| | |
|---|---|
| **Reason for exclusion** | Dublet |

### *Behdani 2013*

| | |
|---|---|
| **Reason for exclusion** | Dublet |

### *Carucci 2017*

| | |
|---|---|
| **Reason for exclusion** | Wrong study design |

### *Carucci 2019*

| | |
|---|---|
| **Reason for exclusion** | Wrong study design |

### *Catala Lopez 2017*

| | |
|---|---|
| **Reason for exclusion** | Wrong study design |

### *Chang 2017*

| | |
|---|---|
| **Reason for exclusion** | Wrong study design |

### *Chang 2018*

| | |
|---|---|
| **Reason for exclusion** | Wrong study design |

### *Chang 2019a*

| | |
|---|---|
| **Reason for exclusion** | Wrong study design |

### *Cooper 2015*

| | |
|---|---|
| **Reason for exclusion** | Wrong patient population |

### *Cooper 2016*

| | |
|---|---|
| **Reason for exclusion** | Wrong patient population |

### *Dean 2014*

| | |
|---|---|
| **Reason for exclusion** | Wrong patient population |

### *Derbyshire 2017*

| | |
|---|---|
| **Reason for exclusion** | Wrong study design |

### *Derbyshire 2017a*

| | |
|---|---|
| **Reason for exclusion** | Wrong study design |

### Fallah 2018

| | |
|---|---|
| Reason for exclusion | Wrong patient population |

### Fibert 2018

| | |
|---|---|
| Reason for exclusion | Wrong study design |

### Foley 2013

| | |
|---|---|
| Reason for exclusion | Wrong study design |

### Ghanizadeh 2015

| | |
|---|---|
| Reason for exclusion | Wrong intervention |

### Goode 2018

| | |
|---|---|
| Reason for exclusion | Wrong study design |

### Gow 2014

| | |
|---|---|
| Reason for exclusion | Wrong study design |

### Gow 2015

| | |
|---|---|
| Reason for exclusion | Wrong study design |

### Hawkey 2014

| | |
|---|---|
| Reason for exclusion | Wrong study design |

### Johnson 2017

| | |
|---|---|
| Reason for exclusion | Wrong patient population |

### Kean 2017a

| | |
|---|---|
| Reason for exclusion | Wrong patient population |

### Kotting 2013

| | |
|---|---|
| Reason for exclusion | Wrong intervention |

### Lange 2015

| | |
|---|---|
| Reason for exclusion | Wrong study design |

### LimAshworth 2013

| | |
|---|---|
| Reason for exclusion | Wrong study design |

### Liu 2015

| | |
|---|---|
| Reason for exclusion | Wrong indication |

### McNamara 2017

| | |
|---|---|
| Reason for exclusion | Wrong study design |

### Moghaddam 2017

| | |
|---|---|
| Reason for exclusion | Wrong outcomes |

### NLimAshworth 2013

| | |
|---|---|
| Reason for exclusion | Wrong study design |

### Pelsser 2017

| | |
|---|---|
| Reason for exclusion | Wrong study design |

### Pelsser 2017a

| | |
|---|---|
| **Reason for exclusion** | Wrong study design |

### Puri 2014

| | |
|---|---|
| **Reason for exclusion** | Wrong study design |

### Ramakrishnan 2016

| | |
|---|---|
| **Reason for exclusion** | Wrong patient population |

### Reading 2013

| | |
|---|---|
| **Reason for exclusion** | Wrong study design |

### Rosi 2020

| | |
|---|---|
| **Reason for exclusion** | Wrong study design |

### Sanna 2019

| | |
|---|---|
| **Reason for exclusion** | Wrong study design |

### Smuts 2013

| | |
|---|---|
| **Reason for exclusion** | Wrong study design |

### Stevenson 2014

| | |
|---|---|
| **Reason for exclusion** | Dublet |

### Tan 2014

| | |
|---|---|
| **Reason for exclusion** | Wrong study design |

### vanderWurff 2017

| | |
|---|---|
| **Reason for exclusion** | Wrong study design |

### Wilens 2017

| | |
|---|---|
| **Reason for exclusion** | Wrong study design |

### Wu 2015

| | |
|---|---|
| **Reason for exclusion** | Wrong outcomes |

*Footnotes*
