# Supplementary material for: Efficacy and Safety of Polyunsaturated Fatty Acids Supplementation in the Treatment of Attention Deficit Hyperactivity Disorder (ADHD) in Children and Adolescents: A Systematic Review and Meta-Analysis of Clinical Trials
Source: Nutrients. 2021 Apr 8;13(4):1226. doi: 10.3390/nu13041226 (PMC8068201; doi:10.3390/nu13041226)
Supplement: Supplementary file 1 [file nutrients-13-01226-s001.zip › Table S4,S5,S6.docx]

**Table S4.** Study identification of the included RCTs.

| **Study’s First**  **Author, Year** | **Region, Country** | **Trial**  **Registration** | **Study Design** | **Conflict of**  **Interest/Sponsorship** |
| --- | --- | --- | --- | --- |
| Aman 1987 | Auckland, New Zealand | Not reported | **Study design:** Randomized controlled trial  **Study grouping:** Crossover | Not reported |
| Arnold 1989 | Columbus, Ohio, USA | Not reported | **Study design:** Randomized controlled trial  **Study grouping:** Crossover | Not reported |
| Assarah 2017 | Tehran, Iran | IRCT138803122000N1 | **Study design:** Randomized controlled trial  **Study grouping:** Parallel group | This work was supported in part by the grant from the Behavioral Sciences Research Center of Shahid Beheshti University of Medical Sciences (Tehran, Iran). |
| Barragan 2017 | Mexico, Mexico | Not reported | **Study design:** Randomized controlled trial  **Study grouping:** Parallel group | The author(s) disclosed receipt of the following financial support for the research, authorship, and/or publication of this article: Manfred Döpfner has received consulting income and research support from Lilly, Medice, Shire, Janssen Cilag, Novartis, and Vifor, and research support from the German Research Foundation, German Ministry of Education and Research, and German Ministry of Health. He has received royalties from books and psychological tests published by Guilford, Hogrefe, Beltz, and Huber. Eduardo Barragán has received consulting income and research support from Lilly, UCB, Cilag, Novartis and Vifor, and research support from the Universidad Nacional Autónoma de México (UNAM). |
| Belanger 2009 | Montréal, Québec | Not reported | **Study design:** Randomized controlled trial  **Study grouping:** Crossover | The present work was supported  by JA DeSève Research Chair in Nutrition (EL) and  NutriSanté Inc (Canada) (financial support and gift of n-3  and n-6 capsules). The authors thank the patients who participated in the trial and are grateful to G Beauséjour and  C Rousseau for their technical help; M Beauchemin, L Chaib,  ME Doucet, I Pelletier and E Tremblay for neurological statements; H Paquette and L Lortie for data collection; I Fortier  for statistical supervising; and D Lebel, M Martel and D Fortier  for their assistance. |
| Bos 2015 | Utrecht, The Netherlands | NCT01554462 | **Study design:** Randomized controlled trial  **Study grouping:** Parallel group | Marco Hoeksma and Ans Eilander are employees of Unilever. The other authors declare no conflict of interest. |
| Chang 2019 | Taichung, Taiwan | CMUH 104-REC-058 | **Study design:** Randomized controlled trial  **Study grouping:** Parallel group | Dr. J.P.-C.C. and Dr. K.P.S. are supported by the following Grants: MOST 108-2320-B-039-048; 108-2314-B-039-016; and 107-2314-B-039-005 from the Ministry of Science and Technology, Taiwan; NHRI-EX108-10528NI from the National Health Research Institutes, Taiwan; and CMU106-S-33, CRS-106-063,DMR-107-202, DMR-107-204, DMR-107-091, DRM-107-097, DRM-108-091, CRS-108-048, CMU108-SR-106, DMR-108-216, CMRC-CMA-3 and Chinese Medicine Research Center from the China Medical University, Taichung, Taiwan. Dr. J.P.-C.C. is supported by a Federation for Women Graduates (FfWG) Main Foundation Grant (2018-2019), UK. Dr. C.M.P. and Dr. V.M. are also supported by the grants “Immunopsychiatry: a consortium to test the opportunity for immune therapeutics in psychiatry’(MR/L014815/1) and ‘Persistent Fatigue Induced by Interferon-alpha: A New Immunological Model for Chronic Fatigue Syndrome ’(MR/J002739/1), from the Medical Research Council (UK), and by the National Institute for Health Research Mental Health Biomedical Research Centre in Mental Health at South London and Maudsley NHS Foundation Trustand King’s College London. |
| Cornu 2018 | Bron, France | NCT00770627 | **Study design:** Randomized controlled trial  **Study grouping:** Parallel group | The study was sponsored by the URGO laboratories. The sponsor had a role in the study design. The study was conducted, analysed and the article was written independently from the funding entity. |
| Crippa 2019 | Lecco, Italy | NCT01796262 | **Study design:** Randomized controlled trial  **Study grouping:** Parallel group | The authors declare that they have no conflict of interest.  The authors disclosed receipt of the following financial support for the research, authorship, and/or publication of this article:  this work was supported by unrestricted research grant from Dietetic Metabolic Food srl., which further provided the investigational product and respective placebo. Funders have not been involved in study design, data collection or analysis, or publication decisions. |
| Dashti 2014 | Yazd, Iran | IRCT201304035393N3 | **Study design:** Randomized controlled trial  **Study grouping:** Parallel group | Not reported |
| Dopfner 2019 | Cologne, Germany | NCT01795040 | **Study design:** Randomized controlled trial  **Study grouping:** Parallel group | The author(s) disclosed receipt of the following financial support for the research, authorship, and/or publication of this article: The study was funded by Vifor Pharma (unrestricted grant) |
| Dubnov Raz 2014 | Tel Hashomer, Israel | Not reported | **Study design:** Randomized controlled trial  **Study grouping:** Parallel group | The oil supplement and placebo were kindly supplied by Magnetika Ltd., Israel. The study was funded by the Israeli Association of Ambulatory Pediatrics. The funding source had no effect on study conduction, data collection, analyses or interpretation, and  on the decision to publish the paper. |
| Gustafsson 2010 | Linköping, Uppsala, Stockholm (two sites), Örebro, Jönkö -  ping, Kalmar and Mariestad, Sweden | Not reported | **Study design:** Randomized controlled trial  **Study grouping:** Parallel group | Sponsors: Hela Pharma AB, Minami Nutrition,  Qb-tech medical engineering company and the Medical  Research Council of Southeast Sweden (FORSS-5539) |
| Hirayama 2004 | Toyama, Japan | Not reported | **Study design:** Randomized controlled trial  **Study grouping:** Parallel group | This work was partly supported by a grant from Japan  Fisheries Association and Foundation for Total Health  Promotion. |
| Hirayama 2014 | Okayama, Japan | Not reported | **Study design:** Randomized controlled trial  **Study grouping:** Parallel group | RR, TI and TH work for companies that distribute  phosphatidylserine. All of the other authors declare that  they have not conflicts of interest.  The study was funded by the Lipamin Foundation,  Tokyo, Japan. |
| Johnson 2009 | Göteborg, Sweden | Not reported | **Study design:** Randomized controlled trial  **Study grouping:** Crossover | Not reported |
| Kean 2017 | Melbourne, Australia | ANZCTRN12610000978066 | **Study design:** Randomized controlled trial  **Study grouping:** Parallel group | The study was funded by a grant to Prof. C. Stough from Pharmalink PtyLtd |
| Manor 2011, Manor 2013 | Petach-Tiqva, Israel | NCT00418184 | **Study design:** Randomized controlled trial  **Study grouping:** Parallel group | I.M., A.M., D.K., S.R., H.T., A.W. declare that they have no conflicts of interest concerning this article.  T.C., Y.R., D.Z.-R., Y.M.: are employees of Enzymotec Ltd. |
| Milte 2015 | Melbourne, Victoria, Australia | ACTRN12607000332426 | **Study design:** Randomized controlled trial  **Study grouping:** Crossover | Funding for this work was received from the Australian Research Council Linkage grant LP0776922 in partnership with Novasel Australia |
| Moghaddam 2017 | Zahedan, Iran | IRCT2015092724209N2. | **Study design:** Randomized controlled trial  **Study grouping:** Parallel group | There is no conflict of interest to be declared. |
| Mohammadzadeh 2019 | Sanandaj, Iran | IRCT2016060128182N2 | **Study design:** Randomized controlled trial  **Study grouping:** Parallel group | No potential conflict of interest relevant to this article was reported. |
| Perera 2012 | Colombo, Sri Lanka | Sri Lanka Clinical Trials Registry: SLCTR/2009/006 | **Study design:** Randomized controlled trial  **Study grouping:** Parallel group | The authors declared no potential conflicts of interest with respect to the research, authorship, and/or publication of this article.  The authors disclosed receipt of the following financial support for the research, authorship, and/or publication of this article: The preparation  of study material was sponsored by Igennus Ltd, Cambridge, UK /  Gpristine Pvt Ltd, Sri Lanka. |
| Raz 2009 | Hadera, Israel. | Not reported | **Study design:** Randomized controlled trial  **Study grouping:** Parallel group | Dr. Raz, Professor Carasso, Professor Yehuda have no  conflicts of interest or financial ties to report.  We wish to thank the TOVA research foundation for providing the TOVA kit for the study. |
| Rodriguez 2019 | Oviedo, Spain | EudraCT trial number 2017–000866-31 for the Sponsor’s  Protocol code number TDAH-OVIEDO. | **Study design:** Randomized controlled trial  **Study grouping:** Parallel group | The authors report no conflicts of interest in this work. |
| Salehi 2016 | Tehran, Iran | IRCT20110416201N1 | **Study design:** Randomized controlled trial  **Study grouping:** Parallel group | There are no conflicts of interest. |
| Sinn 2007 | Adelaide, South Australia | Not reported | **Study design:** Randomized controlled trial  **Study grouping:** Crossover | This study was made possible with the support of the University of  South Australia and CSIRO Human Nutrition, the generous supply of eye qTM supplements by Equazen (London UK) and Novasel (Queensland Australia), and multivitamins and minerals by Blackmores Australia; Channel 10 News, participants, parents, teachers, and schools. |
| Stevens 2003 | Indiana, USA | Not reported | **Study design:** Randomized controlled trial  **Study grouping:** Parallel group | This trial was funded by grants from the National Institute of Mental  Health (# RO3 MH56414) and from Scotia Pharmaceuticals, Ltd.  Wen Zhang and Anne Mahon were supported in part by a grant from  the National Fisheries Institute. |
| Vaisman 2008 | Tel-Aviv, Israel | NCT00382616 | **Study design:** Randomized controlled trial  **Study grouping:** Parallel group | NV is a consultant at Enzymotec  1178 VAISMAN ET AL  by guest on July 4, 2017 ajcn.nutrition.org Downloaded from LTD and DP is the Director of Clinical Studies at Enzymotec LTD. The other authors had no personal or financial conflict of interest. |
| Voigt 2001 | Houston, Texas | Not reported | **Study design:** Randomized controlled trial  **Study grouping:** Parallel group | The study has been funded in part with federal funds from the US Department of Agriculture, Agricultural Research Service, under Cooperative Agreement No. 38-  6250-1-003. The contents of this publication do not necessarily reflect the views or policies of the  US Department of Agriculture, nor does the mention of trade names, commercial products, or  organizations imply endorsement by the United States Government. This study was also funded  in part by a grant from the Martek Biosciences Corporation, Columbia, Maryland, which, in addition, provided the docosahexaenoic acid (DHA) and placebo capsules used in the study |
| Widenhorn Muller 2014 | Ulm, German | NCT 01055119 | **Study design:** Randomized controlled trial  **Study grouping:** Parallel group | Supplement and placebo were kindly provided by Merck  Selbstmedikation (Darmstadt, Germany). Merck Selbstmedikation  had no influence on design or realization of the intervention study.  Data analysis and publication of the trial is the sole responsibility  of the authors. |

**Table S5.** The baseline characteristic of the included RCTs.

| **Study’s First**  **Author, Year** | **N**  **(% Female)** | **Age**  **(Mean years _ SD)**  **(Median years (IQR))** | **Inclusion Criteria** | **Exclusion Criteria** |
| --- | --- | --- | --- | --- |
| Aman 1987 | 31  (13%) | 8.86 (SD 1.88) | Children were admitted if their scores on both the Attention Problem subscale (III)of the RBPC and the inattention subscale (II) of the teacher Questionnaire exceeded the 90^th^ percentile using normative data from Auckland children. | Not reported |
| Arnold 1989 | 18  (0%) | mean and median 9 years | All subjects met the following criteria: (1) age between 6 and 12 years (mean and median 9  years), (2) normal intelligence, (3) diagnosis of attention deficit disorder with hyperactivity  by DSM-III criteria, (4) score of 18 or more on the Conners Hyperactivity Index (Goyette  et al. 1978), (5) sum of 24 or more on the first 6 items of the Davids Hyperkinetic Rating  Scale (Davids 1971), (6) no psychoactive drug in the preceding week, and (7) no history of seizures. | Not reported |
| Assarah 2017 | 40  (33%) | 9.1 (SD 2) | ADHD diagnosis was confirmed based on DSM-IV (4th ed.; American Psychiatric Association, 1994) criteria. All included patients also scored more than 20 based on the Parent ADHD Rating Scale. | Patients with any psychiatric disorder, except for oppositional defiant disorder (ODD) and learning disability (LD), based on Kiddi Scheduled for Affective Disorders Schizophrenia (K-SADS) questionnaire as well as those with intelligence quotient (IQ) less than 70; use of any psychotropic substance, opioid, or other drugs affecting central nervous system in two previous weeks; any significant neurologic disease; and use of any combination containing PUFAs more than once weekly were excluded from the study. |
| Barragan 2017 | 90  (33%) | 8.27 (SD 1.74) | They were required to be 6 to 12 years of age with newly diagnosed ADHD of any subtype (diagnosed according to the Diagnostic and Statistical Manual of Mental Disorders [4th ed., text rev.; DSM-IV-TR]; American Psychiatric Association, 2000). The presence of any associated condition (comorbidity) was assessed by clinical inter-view according to DSM-IV-TR criteria, and the investigator also made a medical evaluation, including weight, height, cephalic perimeter, neurologic evaluation, sleep patterns, and other symptoms or signs. Written informed consent to participate was given by the parents or tutors, and the trial was approved by the local ethical review board | Exclusion criteria included neurologic disorders (epilepsy, brain damage, mental retardation), autism or pervasive developmental disorders, known hypersensitivity to components of Omega-3/6, previous pharmacological treatment for ADHD, ongoing chronic conditions (e.g., asthma), or medication for chronic conditions. Children not receiv-ing school assistance were also excluded from the study. |
| Belanger 2009 | 37  (31%) | PUFA group: 9.27 (SD 0.40)  Placebo: 9.09 (SD 0.50) | The children were between six years  11 months of age and 11 years 11 months of age, with a  Diagnostic and Statistical Manual of Mental Disorders, Fourth  edition (DSM-IV) diagnosis of ADHD based on the results of  the parent and teacher Conners’ questionnaires and a clinical evaluation.  Furthermore, subjects had an IQ score above 85, as evaluated by administering the Wechsler  Intelligence Scale for Children, third edition. The parents of  the subjects agreed to provide three blood samples from their  children during the study. | Subjects diagnosed with mental health disorders (excluding those with characteristic comorbidity associated with ADHD), such as depression, anxiety, tic disorder, conduct disorder, specific learning disabilities and children receiving one or more of the medications, including a psychostimulant or nonstimulant (eg, atomoxetine), sedatives, anxiolytics and antipsychotics, were not included in the present study. Subjects with a medical condition requiring longterm treatment (leukemia or cancer), a chronic neurological condition (cerebral palsy or a metabolic disease) or a paroxysmal disorder (epilepsy); those with allergy to sunflower oil (contained in the placebo) or to fish (the active ingredient in the experimental treatment); and those with coagulation abnormalities, who were candidates for surgery during the duration of the study, or who received anticoagulants,  were also excluded. Also, if more than one child in the same  family had a diagnosis of ADHD, only one child was eligible  to participate in the study and this child was selected at  random. Subjects who consumed natural medicine products  have an increased risk of hemorrhage, and those in whom a surgical procedure was planned were excluded. Patients who  were found to consume fish, flaxseed oil and foods enriched  with n-3 PUFA (eggs, or milk containing n-3 PUFA supplements) during the course of the study were also excluded. |
| Bos 2015 | 40  (0%) | 10.3 (SD 2.0) | The clinical diagnosis  was confirmed by a trained researcher using the Diagnostic  Interview Schedule for Children–Parent Version (DISC-P).  We chose to only include boys in this study as we wanted to  minimize the number of potential confounds (such as gender)  on brain activity, and ADHD is more prevalent in boys than  girls. The children with ADHD were either medication naive  or using psychostimulant medication (methylphenidate only).  Children with ADHD who were on  stimulant medication were instructed not to take their medication  for 24 h before the fMRI scan. However, children were  allowed to use their medication throughout the intervention  period (16 weeks). Medication continued to be managed by  the outside provider (eg, general practitioner, pediatrician, or  psychiatrist). Any changes in medication status were recorded  on a monthly basis by the research team. | No other forms of psychoactive medication were accepted  in this study. |
| Chang 2019 | 92  (14%) | 9.49 (SD 3.05) | The Institutional Review Board of China Medical University Hospital (CMUH, Taichung, Taiwan) approved this study (CMUH 104-REC-058) and written informed consent was obtained from the participants and their parents. We recruited youth aged 6–18 years, with the Diagnostic and Statistical Manual of Mental Disorders, Fifth Edition (DSM-5) diagnosis of ADHD with either inattention (attention deficit disorder, ADD), hyper-activity, or combined presentation; we also assessed the presence of oppositional defiant disorder (ODD), characterised by symptoms such as often losing temper, arguing with adults, defying rules and blaming others. All diagnoses were confirmed by a child and adolescent psychiatrist, and all participants were referred to the Department of Psychiatry, CMUH, from July 2016 to December 2017. The participants were either drug naïve or had no medication for the past 6 months. | The exclusion criteria were: (1) Intelligence quotient<70, based on a documented history of mental retardation; (2) for those ages 6–12 years old, a Peabody Picture Vocabulary Test-Revised (PPVT-R) percentile scores less than 5% (indicating speech delay or intellectual disability);(3) other comorbid psychiatric disorders, such as autism spectrum disorder, anxiety disorder, conduct disorder, and other major psychiatric disorders; (4) comorbid physical disorders, such as thyroid dysfunction and cerebral palsy; (5) currently using n-3 PUFAs supplements; and (6) allergy to n-3 PUFAs. |
| Cornu 2018 | 162  (22%) | 9.90 (SD 2.62) | The study population included children and adolescents aged 6–15 years referred for hyperactivity symptoms to five reference centres for learning disabilities in France. ADHD diagnosis was performed by child psychiatrists specialised in ADHD according to DSM-IV-TR criteria. Briefly, children had to have at least six hyperactivity–impulsivity symptoms for six months or more, and/or at least one of six inattention symptoms for six months or more; certain symptoms had to be present before the age of 7 years, and there was a functional impairment in two or more environment (school, home), with a clinically significant alteration in the social, school, or family functioning. Symptoms had not to be part of another psychiatric disorder | Exclusion criteria were: known intolerance to omega-3 fatty acids, intake of fatty acid/fish oil dietary supplements for more than 1 week during the 3 months preceding inclusion, or MPH or other ADHD drug during the month preceding inclusion. Children who required MPH treatment were also excluded to ensure equipoise. |
| Crippa 2019 | 46  (9%) | PUFA: 11.06 (SD 1.85)  Placebo: 10.91 (SD 1.42) | Participants aged 7 to 14 were recruited from the Child Psy-chopathology Unit at our institute over a 22-month period. The study coordinator contacted 128 parents by phone to invite children to participate in the study protocol. Of these, 50 children with ADHD and their parents agreed to participate. The main reason for declining to participate was a child’s refusal to have his or her blood sampled. All participants were diagnosed by a child neuropsychiatrist in accordance with the diagnostic and statistical manual of mental disorders criteria (fourth ed., text rev.; [15]). A child psychologist experienced in the diagnosis of ADHD (AC) confirmed independently the diagnosed by through direct observation and the administration of the semi-structured interview Development and Well-Being Assessment (DAWBA; [16]). According to the clinical assessment, 15.7% of children met the criteria for the ADHD inattentive subtype, 33.3% fulfilled criteria for the hyperactive–impulsive subtype, and 51% had the combined subtype. The Wechsler Intelligence Scale for Children–III or –IV [17, 18] was used to obtain the Full Scale Intelligence Quotient (FSIQ) or FSIQ scores. Only participants with FSIQ or esti-mated FSIQ scores higher than 80 were included. Moreover, all children were required to be drug-naïve and not have consumed omega-3/omega-6 supplements during the 3 months prior to the recruitment. | Exclusion criteria were a history of seizures, other neurological disorders, or diagnosed genetic disorders. All participants were Caucasian and had normal or corrected-to-normal vision |
| Dashti 2014 | 56  (45%) | 8.22 (SD 1.65) | Children with a score of higher than 65 on both scales, IQ of higher than 70 using the Kaufman Brief Intelligence Test, and whose parents gave an informed consent were included. | However, children with occurrence of unintended side effect of methylphenidate, who had previous treatment of ADHD, who had taken omega-3 supplement during the previous 3 months, and who had severe psychiatric disorders were excluded from the study. |
| Dopfner 2019 | 40  30% | 5.55 years (SD 0.61) | Children were eligible for the study if they were aged 3 to 6 years, if their parents or preschool teachers reported that they had elevated levels of ADHD symptoms, if they were not receiving any current medication for ADHD, and if their parents spoke German. Moreover, chil-dren who were already receiving a treatment aiming at the reduction of behavior problems were included in the study if no change of treatment was planned. | Exclusion criteria were a hypersensitivity to components of the study product (fish oil, primrose oil, natural strawberry flavoring, or bovine gelatin), the consumption of an omega fatty acids preparation, or the consumption of fish oil capsules before the start of the study |
| Dubnov Raz 2014 | 17  (41%) | Sage oil: 11.1 (SD 3.0)  Placebo: 10.9 (SD 2.3) | The study population included 40 children and adolescents aged6–16 years, recently diagnosed with ADHD, who were drug naïve and untreated, from two ambulatory ADHD specialty clinics in Israel. | Exclusion criteria were refusal to undergo any or all of the testing procedures or to take the designated supplement; a history of chronic health conditions other than ADHD; or use of any chronic medications or dietary supplements, specifically methylphenidate or fatty acid/fish oil supplements. |
| Gustafsson 2010 | 92  (17%) | 7–12 years  (mean data not reported) | Children between 7 and 12 years of age with a clinical diagnosis of ADHD of combined type (fulfilling DSM-IV criteria A–E) with any neuropsychiatric co-morbidity, and who had been evaluated for pharmacological treatment, were allegeable for participation in this prospective, randomized and  double-blinded study. In Sweden, pharmacologic treatment  of ADHD normally is initiated at specialist level. Co-morbidity was evaluated in the following ways: oppositional behaviour with CTRS Oppositionality subscale; neuromotor problems (DCD) with a neuromotor examination (32);  objective hyperactivity ⁄ impulsivity was measured with the continuous performance computerized test (see below); and tics, anxiety and Asperger syndrome by clinical interview.  Patients were included if patient and family were able  to follow the study protocol and adhere to it, and had no  medical conditions requiring intervention. | Exclusion criteria were mental retardation (IQ < 70 on formal psychological testing), autism, major depression, epileptic seizure (including Petit Mal) during the proceeding 2 years, other neurological disorder, other endocrinological disorders (i.e. diabetes mellitus, thyroid disorder, etc.), fish allergy,  severely impaired hearing and vision, severe sleeping disorder, psychotic symptoms or other ongoing medication (i.e.  psychoactive compounds, anti-convulsants, stimulants). |
| Hirayama 2004 | 40  (20%) | DHA group: 9 (6.8, 11.3)  Control group: 9 (7, 10.3) | A total of 40 school children (6–12 y of age) were recruited  for the present study from a summer camp for children with  psychiatric disorders run by one of the authors (SH). The  summer camp consisted of 68 children of 3–15 y of age,  about 90% of whom suffered from AD/HD as the major  disorder. In this group, there were 46 AD/HD children of  6–12 y of age. We asked parents of those 46 children to  participate in the present study, and 40 of them agreed  (Figure 1). These 40 subjects had been diagnosed or  suspected as AD/HD according to DSM-IV and diagnostic  interviews including behavior observation by psychiatrists.  In a strict sense, eight subjects might not be AD/HD  according to the DSM-IV criteria, but two psychiatrists  attending the summer camp strongly suspected them as  AD/HD. | Not reported |
| Hirayama 2014 | 36  (5%) | PUFA: 9.1 (SD 1.7)  Placebo: 8.7 (SD 3.0) | Patients with ADHD who had not received any drug treatment related to ADHD | Not reported |
| Johnson 2009 | 75  (15%) | 12 (SD 2.16) | Patients aged  8 to 18 years who met DSM-IV criteria for a diagnosis of  ADHD of any subtype, scoring at least 1.5 SD above the  age norm for their diagnostic subtype using norms for  the ADHD Rating Scale–IV–Parent Version (ADHDRS-IV; DuPaul, Power, Anastopoulos, & Reid, 1998),  were included in the study. The presence of any associated condition (comorbidity) was assessed by clinical  interview according to DSM-IV criteria. Reading and  writing ability was determined by standardized tests and  RWD diagnosed according to DSM-IV criteria for reading  disorder and/or disorder of written expression (APA,  2000). The term learning difficulties (LD) used in this  article corresponds to the DSM-IV diagnosis of borderline  intellectual functioning. | Exclusion criteria for entry into  the study were autism (however, autistic symptoms [AS]  diagnosed in cases meeting three or more but not full  symptom criteria for a diagnosis of autistic disorder,  Asperger syndrome, or any of the other autism spectrum  disorders was not an exclusion criterion), psychosis,  bipolar disorder, mental retardation, uncontrolled seizure  disorder, hyper- or hypothyroidism, significant other  medical conditions, weight below 20 kg, alcohol or drug  abuse, or the use of any psychoactive drugs or omega 3  preparations in the past 3 months. |
| Kean 2017 | 144  (15%) | 8.7 (SD 2.24) | Inclusion criteria were as follows: healthy, non-smokingmales and females aged between 6 and 14 years, who hadDiagnostic and Statistical Manual of Mental DisordersFourth Edition (DSM-IV) ADHD rating score of greater than15, who were fluent in English, had parental or legal guardianconsent and verbal consent from the child. The DSM-IVratingscale is a reliable and valid four-point (0 = never or rarely,1 = sometimes, 2 = often, 3 = very often) 18-item semi-struc-tured interview that assesses symptom severity (Faries et al.2001). A score of 15 points or higher on the DSM-IVrating scale allowed investigators to establish that partic-ipants had elevated levels of hyperactivity, inattention orboth. Criteria for ADHD subtypes (inattentive or hyperac-tive-impulsive), requires six or more scores in the higherrange (2 = often, 3 = very often) of the scale for thatsubtypes (DuPaul et al.1998). | Exclusion criteria were as follows: primary medical diagnosisother than ADHD, oppositional defiant disorder or similarbehavioural disorders; currently taking any medication (otherthan stimulants if a formal diagnosis of ADHD or other be-havioural disorder has been made); current or history of heartdisease, or high blood pressure, or diabetes; health conditionsthat would affect food metabolism, including the following:food allergies, kidney disease, liver disease and/or gastrointes-tinal diseases (e.g. irritable bowel syndrome, coeliac disease,peptic ulcers); pregnant or breast feeding; unable to participatein all scheduled visits, treatment plan, tests and other trialprocedures according to the protocol; allergy to shellfish; ep-ilepsy or photosensitivity. |
| Manor 2011, Manor 2013 | 147  (PS-Omega 3: 28%, Placebo: 32%) | PS-Omega 3: 9.2 (SD 2.0)  Placebo: 9.2 (SD 1.8) | a) confirmed DSM-IV-ADHD diagnosis following assessment by the Schedule for Affective Disorders and Schizophrenia for  School-Age Children-Present and Lifetime (K-SADS-PL) Version  1 [36];  b) a score of at least 1.5 standard deviations above the normal for  the patient’s age and gender in the Teacher-rated ADHD Rating  Scale-IV (RS-IV) School Version;  c) a score of 4 or higher (moderately ill or worse) in the Clinical Global Impression of Severity of Illness (CGI-S) test;  d) willingness of the parent and a teacher who is familiar with the  child to participate. | a) girls who reached menarche and presented with three previous  regular menstrual cycles;  b) history or current diagnosis of any serious systemic (e.g.,  diabetes, hyper/hypothyroidism) or neurological condition (e.g.,  epilepsy, brain tumor);  c) failure to respond to two or more adequate courses of stimulant  therapy (among those previously treated children);  d) pervasive developmental disorder (diagnosed according to  DSM-IV criteria) or nonverbal learning disability [19,26];  e) diagnosed with psychotic disorders (e.g., schizophrenia)  according to the DSM-IV axis;  f) any evidence of suicidal risk or any current psychiatric  comorbidity that required psychiatric pharmacotherapy;  g) concomitant use of prescription or nonprescription agents with potent psychotropic properties, including ADHD treatments and dietary supplements, 4-week prior to the study entry;  h) history of alcohol or substance abuse as defined by DSM-IV  criteria;  i) consumption of > 250 mg/day of caffeine;  j) history of allergic reactions or sensitivity to marine products,  soy, or corn as well as any illness that could jeopardize the  participant’s health or limit their successful completion of the trial. |
| Milte 2015 | 87  (23%) | 8.91 (SD 1.729) | Children, ADHD diagnosis, parent-reported learning difficulties, willing to fulfil requirements of the study. | Cannot be on stimulant medication, cannot have taken omega-3 supplements for up to 3 months. |
| Moghaddam 2017 | 40  (17%) | 9.5 (SD 2.0) | Inclusion criteria were obtaining the least score in an ADHD rating scale questionnaire, responding to the treatment based on least reduction of 25% of symptoms relative to the base state in ADHD scaling | Exclusion criteria were: Any type of concurrent major psychological disease except oppositional defiant disorder (ODD) and learning disorder (OD), intelligence quotient below 70, taking psychedelic drugs or drugs in two recent weeks, any major neurological disease and taking any drug affecting the nervous system at least two weeks before the study. |
| Mohammadzadeh 2019 | 66  (Methylphenidate and omega-3: 33%, Methylphenidate and placebo: 21%) | Methylphenidate and omega-3: 7.7 (SD 1.65)  Methylphenidate and placebo: 8.20 (SD 1.72) | The study population consisted of 66 children (49 boys and 17 girls) aged 6 to 12 years old who were selected through simple random sampling. The ADHD was diagnosed by a psychiatrist of children and adolescents based on Diagnostic and Statistical Manual of Mental Disorders, Fourth Edition, Text Revision (DSM-IV-TR) criteria. A structured clinical interview with Kiddie Schedule for Affective Disorders and Schizophrenia was used to reject other disorders.Patients were from all ADHD subtypes and new ones. The parents of children described the ADHD symptoms in accordance with the DSM-IV-TR criteria | Children who had a history of supplementa-tion with omega-3 in at least the last 6 months, the presence of any known physical illness, mental disability, known psychiatric disor-ders (autism spectrum disorders, schizophrenia, intellectual impair-ment, and other psychiatric disorders), seizure, any psychiatrist comorbidity which needs treatment and children with suicide ideas were excluded |
| Perera 2012 | 94  (Study Group: 29%, Placebo Group: 24%) | Study Group: 9.4 (SD 1.5)  Placebo Group: 9.2 (SD 1.5) | Participants for the study were children 6 to 12 years of age, selected from an outpatient treatment program for ADHD. All children in the program were clinically diagnosed (according to criteria from the Diagnostic and  Statistical Manual of Mental Disorders, Fourth Edition), supported by positive scores in Swanson, Nolan and Pelham version IV(SNAP) parent  and teacher evaluation. The associated behavior problems and difficulties in academic learning were made on clinical history and teacher report  (based on age-related academic standards set for Sri Lankan children by the Institute of Education, Sri Lanka). All children were prescribed a total daily dose of methylphenidate (0.7-1 mg/kg body weight) and standard behavioral intervention. | Not reported |
| Raz 2009 | 63  (EFA: 41%, Placebo: 39%) | EFA: 10.48 (SD 1.42)  Placebo: 10.51 (SD 1.47) | Inclusion criteria were: subjects should be 7–13 years old and have a written ADHD diagnosis from a child psychiatrist, a neurologist, a pediatrician, or a clinical psychologist. (This diagnosis was made using the standard clinical practice, and not as a part of the trial or by our staff; it was not based on the  assessment in the trial.) | Exclusion criteria were: Use of medications for ADHD during the past month, use of EFA supplements during the past 3 months, or the presence of at least one of the following co-morbidities—pervasive developmental disorder, seizure disorder, schizophrenia, major depression, or bipolar disorder |
| Rodriguez 2019 | 66  (29%) | 11.7 (SD 3.1) | Boys and girls between 6 and 18 years of age with a DSM-5 diagnosis of ADHD (DSM-5 American Psychiatric Association, 2013) were recruited through the Faculty of Psychology at the University of Oviedo. The clinical diagnosis was confirmed by a trained researcher using the Diagnostic Interview Schedule for Children–Parent Version (DISC-P). Patients with any subtype of ADHD (hyperactive-impulsive, inattentive, combined hyperac-tive-inattentive) were eligible. The children with ADHD were either medication naïve or using psychostimulant medication. | Exclusion criteria were blood coagulation dis-orders, cognitive impairment or autism spectrum disorder, known intolerance to fish proteins, and treatment with dietary supplements containingω-6 orω-3 PUFAs during the month preceding inclusion. Participants with extreme total IQ scores lower than 70 and greater than 130 using the Wechsler Intelligence Scales for Children, fourth edi-tion (WISC-IV)31were excluded. |
| Salehi 2016 | 150  (PUFA: 61%, Placebo: 68%) | PUFA: 8.6 (SD 1.7)  Placebo: 9.12 (SD 2.2) | Inclusion criteria were set for children of 6–15 years old, with a recent diagnosis of ADHD based on DSM-IV-TR criteria, no history of psychiatric drug usage and no history of other psychiatric disorders, no limitation or sensitivity for the use of zinc sulfate and omega-3, and absence of mental retardation | Moreover, exclusion criteria included children who develop acute systemic diseases, who cannot use oral medication, who refuse the medication due to side effects, and whose method of treatment has been changed. |
| Sinn 2007 | 132  (20%) | PUFAs + MVM: 9.20 (SD 1.72)  PUFAs: 9.44 (SD 1.90)  Placebo: 9.67 (SD 1.90) | Children were included in the study if they were between the ages of 7 and 12 years and had scores 2 SD above the general population average (above the 90^th^ percentile) on Conners abbreviated ADHD Index,36  which assesses problems with hyperactivity, impulsivity,  and attentional/cognitive problems. | They were excluded  if they were taking any form of stimulant medication and  if they had taken any form of omega-3 supplementation in  the previous 3 months. |
| Stevens 2003 | 47  (PUFA: 11%, Placebo: 13%) | PUFA: 9.5 (SD 1.7)  Placebo: 10.1 (SD 2.0) | Children with AD/HD symptoms were selected for the study only if their total thirst/skin score was 4 or greater. | Participants were excluded from participation in the study for the following reasons: age, distance from the test site, inability to swallow capsules, lack of further interest, or chronic illness (diabetes). |
| Vaisman 2008 | 60  (25%) | Phospholipids enriched with  n-3 fatty acids: 9.17 (SD 1.27)  Fish oil: 9.40 (SD 1.06)  Placebo: 9.31 (SD 1.28) | Children were included if they were 8–13 y of age and had received a previous diagnosis of ADHD by a clinical psychiatrist, neurologist, or pediatrician. | Children with significant sensory or neurological limitations, epilepsy, mental retardation, psychosis, or pervasive developmental disorder were excluded. |
| Voigt 2001 | 54  (DHA group: 22%, Placebo: 22%) | DHA group: 9.1 (SD 2.1)  Placebo: 9.5 (SD 1.7) | The remaining 90 children, all of whom had previously been given a diagnosis of ADHD by a physician and were being treated successfully with stimulant medication, underwent a confirmatory diagnostic interview with a neurodevelopmental pediatrician to confirm responses to the telephone interview and  to ensure that each met strict Diagnostic and Statistical Manual of Mental Disorders (DSM-IV)1 criteria for ADHD and had clear historical evidence of clinically significant impairment in social or academic functioning. Children who met DSM-IV criteria for oppositional defiant disorder or conduct disorder, both of which are common comorbid conditions in children with ADHD, 24 were not excluded. Seventy of the 90 potential subjects who completed the diagnostic interview were considered to have idiopathic ADHD and were offered study enrollment; 63 returned for randomization. | A total of 160 children were  excluded for one or more of the following reasons: (1) ineffective treatment with stimulant medication; (2) treatment with other psychotropic medications; (3) previous diagnosis of other  childhood psychiatric disorders (ie, anxiety, mood, thought, or bipolar disorders), (4) use of dietary supplements  other than vitamins, (5) occurrence of a significant life event (eg, death of an immediate family member, parental  separation or divorce, or relocation) within 6 months; (6) a history of head injury or seizures; (7) receipt of special education services for mental retardation or a pervasive developmental disorder; (8) premature birth; (9) exposure to tobacco, alcohol, or other drugs in utero; and/or (10) diagnosis of a disorder of lipid metabolism or other chronic medical condition. |
| Widenhorn Muller 2014 | 95  (21%) | PUFA: 8.90 (SD 1.48)  Placebo: 8.92 (SD 1.24) | Children of both sexes 6–12 years of age, meeting DSM-IV criteria for the ADHD combined subtype (hyperactive–inattentive), the primarily inattentive or the hyperactive/impulsive subtype were included in the trial. | Exclusion criteria were IQr70, use of stimulant medication and other psychoactive medication as well as fatty acid supplements used within the previous 6 months. Children with allergies against fish or fish products were also excluded from participation. |

**Table S6.** The intervention, control, reported outcomes and authors conclusion of the included RCTs.

| **Study’s First**  **Author, Year** | **Intervention** | **Control** | **Reported outcomes** | **Authors**  **Conclusion** |
| --- | --- | --- | --- | --- |
| Aman 1987 | **Description:** 3 capsules twice daily of essential fatty acids (efamol) per day. Each capsule contained 360 mg of linoleic acid and 45 mg of gamma-linoleic acid.  **Duration (wk):** 4 weeks | **Description:** 3 capsules twice daily of 500 liquid paraffin.  **Duration (wk):** 4 weeks | No relevant outcomes | Minimal or no improvements in hyperactive children. |
| Arnold 1989 | **Description:** Each subject took five capsules each morning and four capsules each afternoon. **Duration:** 3 months.  Dose: One of the morning capsules was either D-amphetamine timed-release spansule. The other eight capsules were either Efamol. Each Efamol capsule  contained 500 mg of evening primrose oil, which supplies 40 mg of GLA and 350 mg  of linoleic acid. Each capsule also contained 13 international units of vitamin E (D-alpha  tocopherol acetate) as a preservative. The D-amphetamine spansule was either 10 mg or  15 mg, selected to provide between 0.35 mg and 0.70 mg D-amphetamine per kilogram  body weight. | **Description:** Each subject took five capsules each morning and four capsules each afternoon.  Matched placebo (liquid paraffin, a hydrocarbon mineral oil containing no fatty acids).  **Duration:** 3 months. | ADHD Core symptoms, teacher rated, end of treatment | This study does not establish Efamol as an effective treatment. |
| Assarah 2017 | **Description:** The intervention group received methylphenidate with the dose of 0.3 mg/kg/day in two divided doses that was increased to 1 mg/kg/day during 2 weeks. The treatment arm also received 430 mg capsules containing 241 mg DHA, 33 mg EPA, and 180 mg omega-6 (Minami Company, Belgium) once daily.  **Duration (wk):** 10 weeks  **Dose:** 430 mg capsules containing 241 mg DHA, 33 mg EPA, and 180 mg omega-6 once daily. | **Description:** The control group received methylphenidate with the dose of 0.3 mg/kg/day in two divided doses that was increased to 1 mg/kg/day during 2 weeks. The control arm received identical placebo capsules from the same com-pany with the same order.  **Duration (wk):** 10 weeks | ADHD Core symptoms, parent rated, end of treatment | The results did not support the efficacy of PUFA in the  treatment of ADHD |
| Barragan 2017 | **Description:** The Omega-3/6 fatty acid supplement used for the trial was Equazen eye q™ (provided by Vifor Pharma, Switzerland). Patients received three capsules twice daily, corresponding to a daily dose of 558 mg EPA, 174 mg DHA, and 60 mg GLA. This product and dosage were chosen based on effi-cacy and tolerability results from previous clinical trials (Johnson, Ostlund, Fransson, Kadesjo, & Gillberg, 2009; Richardson & Montgomery, 2005; Sinn & Bryan, 2007).The MPH preparation used in the study was long-acting Metadate CD® (Shire Pharmaceuticals Mexico SA). Patients in the MPH arm received an initial daily dose of 0.3 mg/kg/day, increased to 0.5 mg/kg/day after the first 2 weeks of the titration period. The dose was subsequently increased to a maximum of 1 mg/kg/day depending on response and toler-ability. Dose adjustments were made weekly.  **Dose:** 558 mg EPA, 174 mg DHA, and 60 mg GLA  **Duration:** 12 months | **Description:** MPH:The MPH preparation used in the study was long-acting Metadate CD® (Shire Pharmaceuticals Mexico SA). Patients in the MPH arm received an initial daily dose of 0.3 mg/kg/day, increased to 0.5 mg/kg/day after the first 2 weeks of the titration period. The dose was subsequently increased to a maximum of 1 mg/kg/day depending on response and tolerability. Dose adjustments were made weekly.  **Dose:** MPH arm received an initial daily dose of 0.3 mg/kg/day, increased to 0.5 mg/kg/day after the first 2 weeks of the titration period. The dose was subsequently increased to a maximum of 1 mg/kg/day depending on response and tolerability. Dose adjustments were made weekly.  **Duration:** 12 months | ADHD Core symptoms, parent rated, end of treatment  Side effects (Nausea, Diarrhea) | The tested combination  of Omega-3/6 fatty acids had similar effects to MPH, whereas the MPH + Omega combination appeared to have some  tolerability benefits over MPH. |
| Belanger 2009 | **Description:** each capsule of the active n-3 PUFA supplement consisted of 25 mg of PL, 250 mg of EPA, 100 mg of  DHA and 3.75 U of alpha-tocopherol (vitamin E). Vitamin E  was added to prevent oxidation of the fatty acids (FA) in the  capsule. The total daily dose of the active n-3 PUFA supplement was administered once or twice per day according to the  body weight of the children. Because the capsules could not be divided, participants weighing 16 kg to 25 kg received two  capsules daily (500 mg EPA), those weighing 26 kg to 35 kg  received three capsules daily (750 mg EPA), and those weighing 36 kg to 45 kg received four capsules daily (1000 mg EPA)  **Duration (wk):** 16 weeks | **Description:** The placebo capsule contained 500 mg of sunflower oil, with no n-3 PUFA, and was composed of 70% linoleic acid (a precursor of n-6 PUFA), 20% oleic acid, and palmitic and stearic  acid (5% each). A similar amount of vitamin E (as per n-3  capsules) was included in the n-6 capsules.  **Duration (wk):** 16 weeks | No relevant outcomes | A subgroup of children with ADHD who used n-3  PUFA supplements achieved and maintained symptom control. The data of the present study also supported n-3 PUFA safety and tolerability, but limited  changes were noted in the FA profile in French Canadians with ADHD |
| Bos 2015 | **Description:** All participants were instructed to consume a  daily dose of 10 g of either normal or omega-3 fortified  margarine. The active product was full fat (80%) margarine,  containing 650 mg DHA and 650 mg EPA per 10 g serving.  The dose of the active ingredients DHA and EPA in the  intervention product was under the US Generally Recognized  As Safe (GRAS) level (FDA, 2004).  **Duration (wk):** 16 weeks | **Description:** The placebo  product was a similar margarine with the same sensory  properties, but with monounsaturated fatty acids (refined plant oils) instead of EPA and DHA; the total amount of  saturated fatty acids and omega-6 fatty acid were matched in  the placebo and active product.  **Duration (wk):** 16 weeks | ADHD Core symptoms, parent rated, end of treatment  Behavioral difficulties, teacher rated, end of treatment | This study shows that dietary supplementation  with omega-3 fatty acids reduces symptoms of ADHD. |
| Chang 2019 | **Description:** One hundred andthree youth were recruited and randomised to n-3 PUFAs(1.2 g/day EPA  **Duration (wk):** 12 weeks | **Description:** placebo (1.2 g/day soybean oil)  **Duration (wk):** 12 weeks | ADHD Core symptoms, parent rated, end of treatment  ADHD Core symptoms, teacher rated, end of treatment  Behavioral difficulties, parent rated, end of treatment  Behavioral difficulties, teacher rated, end of treatment  Side effects (Diarrhea) | EPA treatment  improves cognitive symptoms in ADHD youth, especially if they have a low baseline endogenous EPA level, while  youth with high EPA levels may be negatively affected by this treatment. |
| Cornu 2018 | **Description:** The studied dietary supplement consisted of soft capsules containing fish oil rich in vitamin A, D, and E.  **Dose:** EPA (eicosapentaenoic acid) 336 mg and DHA (docosahexaenoic acid) 84 mg; for children aged 9–11 years, EPA 504 mg and DHA 126 mg, and for chil-dren aged 12–15 years EPA: 672 mg and DHA 168 mg [18]; capsules also contained 100 μg vitamin A, 1.25 μg vitamin D, and 3.5 mg vitamin E.  **Duration:** 3 months | **Description:** Placebo composed of olive oil  **Dose:** They were composed of olive oil, the same amount of vitamin A, D, and E, with traces of marine lipid concentrate: EPA (18%), DHA (12%), totaling 4.83 mg, to give the capsules a similar taste and smell.  **Duration:** 3 months | ADHD Core symptoms, parent rated, end of treatment | This study did not show any beneficial effect  of omega-3 supplement in children with mild ADHD  symptoms. |
| Crippa 2019 | **Description:** DHA (alga oil)  **Dose:** two soft gelatin pearls per day providing a dose of 500 mg algal DHA  **Duration:** 6 months | **Description:** Placebo (weat germ oil)  **Dose:** Placebo treat-ment consisted of two pearls per day containing 500 mg wheat germ oil. The placebo was stabilized with a low con-centration of Vitamin E.  **Duration:** 6 months | ADHD Core symptoms, parent rated, end of treatment  Behavioral difficulties, parent rated, end of treatment  Quality of Life, longest follow-up | The 6 months treatment  with supplemental DHA appears to have small positive effects on other behavioral and cognitive difficulties, which, in light  of the absence of side-effects, could be reasonably followed up in future intervention studies. ( |
| Dashti 2014 | **Description:** Omega-3  **Dose:** 1g per day  **Duration (wk):** 4 weeks (not stated in article, taken from protocol) | **Description:** Placebo  **Duration (wk):** 4 weeks (not stated in article, taken from protocol) | ADHD Core symptoms, parent rated, end of treatment | Omega-3 has significantly impacted both groups of hyperactivity-impulsivity and combined  type. |
| Dopfner 2019 | **Description:** Verum group participants received a 4-month treatment with two capsules of an Omega-3/Omega-6 fatty acid sup-plement twice daily, corresponding to a daily dose of 372 mg EPA, 116 mg DHA, and 40 mg GLA. The supplement used for the trial was Equazen Eye Q™ (provided by Vifor Pharma, Switzerland). This product and the ratio of the dif-ferent fatty acids were chosen based on efficacy and toler-ability results from previous clinical trials  **Dose:** two capsules of an Omega-3/Omega-6 fatty acid sup-plement twice daily, corresponding to a daily dose of 372 mg EPA, 116 mg DHA, and 40 mg GLA.  **Duration:** 4 months | **Description:** Placebo group participants received four capsules of a placebo without Omega-3/Omega-6 fatty acids daily  **Duration:** 4 months | ADHD Core symptoms, parent rated, end of treatment  ADHD Core symptoms, teacher rated, end of treatment | The intention-to-treat analyses  provide some evidence for positive effects of Omega-3/Omega-6 fatty acids. |
| Dubnov Raz 2014 | **Description:** Sage oil. The composition of sage oil varies slightly by crop year,and is 50–54% ALA, 20–23% oleic acid, 16–18% linoleic acid, 6–7% palmitic acid, and 2–3% stearic acid  **Dose:** 2g per day  **Duration (wk):** 8 weeks | **Description:** lactose placebo in gel capsules  **Duration (wk):** 8 weeks | ADHD Core symptoms, parent rated, end of treatment  ADHD Core symptoms, teacher rated, end of treatment | Supplementation of 2 g/day of oil containing 1 g ALA did not significantly  reduce symptoms in children with ADHD. |
| Gustafsson 2010 | **Description:** one capsule daily by oral administration of PlusEPA (Minami Nutrition, Antwerp,  Belgium).  **Dose:** One capsule PlusEPA contained 500 mg EPA + 2.7 mg DHA and 10 mg Vitamin E mixed  tocopheroles.  **Duration (wk):** 15 weeks | **Description:** The placebo was a mixture of rape seed oil and medium-chain triglycerides contained in a capsule identical to the one used for PlusEPA. It contained <10% of the PlusEPA content of omega-3 LCPUFA.  **Duration (wk):** 15 weeks | ADHD Core symptoms, parent rated, end of treatment  ADHD Core symptoms, teacher rated, end of treatment  Behavioral difficulties, parent rated, end of treatment  Behavioral difficulties, teacher rated, end of treatment  Side effects (Diarrhea, Nausea) | In this double-blind RCT study, two subgroups of children  with ADHD, characterized by oppositional behaviour and  less hyperactivity ⁄ impulsivity, responded with significant  reduction of the ADHD symptoms by supplementation with  EPA. |
| Hirayama 2004 | **Description:** Subjects in the DHA group took fermented soybean milk (600 mg DHA/125 ml, 3/week), bread rolls (300 mg DHA/45 g, 2/week) and steamed bread (600 mg DHA/60 g, 2/week). As a total, their average intake of n-3 fatty  acids from those foods was 3600 mg DHA þ 700 mg eicosapentaenoic acid (EPA)/week.  **Duration:** 2 months | **Description**: Those in the control group took indistinguishable placebo foods containing olive oil instead  of DHA-rich fish oil.  **Duration:** 2 months | No relevant outcomes | DHA supplementation did not improve AD/HD-related symptoms. Treatment of ADHD with fatty acids deserves  further investigation, but careful attention should be paid as to which fatty acid(s) is used. |
| Hirayama 2014 | **Description:** For this study, cocoa-flavored chews were used, containing 100 mg of soy-derived PS (Phospholipid) per chewable.  **Duration (wk):** 8 weeks  **Dose:** 200 mg of soy-derived PS per day for 2 months. | **Description:** Placebo chews  **Duration (wk):** 8 weeks | ADHD Core symptoms, parent rated, end of treatment  Side effects (Diarrhea, Nausea, gastrointestinal discomfort) | PS significantly improved ADHD symptoms and short-term  auditory memory in children. PS supplementation might be a safe and natural nutritional strategy for improving mental performance in young children suffering from ADHD. |
| Johnson 2009 | **Description:** At the start of Study Period 1, the patients  were randomized to active treatment with omega 3/6 in a  dose of three capsules twice daily, corresponding to a daily  dose of 558 mg EPA, 174 mg DHA (both are omega-3  fatty acids), 60 mg gamma linoleic acid (an omega 6  fatty acid), and 10.8 mg Vitamin E.  **Duration:** 6 months | **Description:** placebo (identical capsules containing olive oil)  **Duration:** 6 months | No relevant outcomes | A subgroup of children and adolescents with ADHD, characterized by inattention and associated neurodevelopmental disorders, treated with omega 3/6  fatty acids for 6 months responded with meaningful reduction of ADHD symptoms |
| Kean 2017 | **Description:** The active trial treatment was the naturally occurringomega-3 anti-inflammatory extract PCSO-524®. The lipidextract PCSO-524® of the New Zealand green-lipped mus-sel is marketed under the brand names Lyprinol® andOmega XL®. The principal ingredients per 260 mg capsulefor the active capsules include PCSO-524® GLM pat.lipids (eicosatetraenoic acid)—50 mg (including EPA7.3 mg and DHA 5.5 mg, natural mono-unsaturated oliveoil 100 mg and vitamin E (D-alpha-tocepherol) as anPsychopharmacology (2017) 234:403–420405 antioxidant 0.225 mg). PCSO-524® also includes sterolesters that consist of mainly myristic acid, palmitic acid,palmitoleic acid, stearic acid, oleic acid and linoleic acid.The sterols found in this fraction included cholesterol,cholesta-3,5-diene, 26,27-dinoergostadienol, cholesta-5,22-dien-3-ol and ergosta-5,22-dien-3-ol.  **Duration (wk):** 14 weeks | **Description:** The placebocapsule contained 35.5 mg of olive oil, 112 mg of lecithin,12 mg of coconut oil and 0.5 mg of 30% beta-carotene.Both treatments were contained within capsules thatconsisted of gelatin, sorbitol syrup and glycerin. The place-bo capsule matched the PCSO-524® capsule in touch, taste,smell and size.  **Duration (wk):** 14 weeks | ADHD Core symptoms, parent rated, end of treatment  Behavioral difficulties, parent rated, end of treatment | The results presented indicate that PCSO-524®  may be beneficial in reducing levels of hyperactivity and inattention in a population of children with clinical and subclinical symptoms of ADHD |
| Manor 2011, Manor 2013 | **Description:** 300mg PS and 120mg EPA + DHA  **Dose:** 420 mg  **Duration (wk):** 15 weeks | **Description:** Cellulose  **Duration (wk):** 15 weeks | ADHD Core symptoms, parent rated, end of treatment  ADHD Core symptoms, teacher rated, end of treatment  Behavioral difficulties, parent rated, end of treatment  Behavioral difficulties, teacher rated, end of treatment  Quality of life, Longest follow-up  Side effects (Diarrhea, Nausea, gastrointestinal discomfort) | The results of this 30-week study suggest that PS-Omega3 may reduce ADHD symptoms in  children. Preliminary analysis suggests that this treatment may be especially effective in a subgroup of  hyperactive-impulsive, emotionally and behaviorally-dysregulated ADHD children. |
| Milte 2015 | **Description:** EPA-rich fish oil, providing a total of 1.109 mg EPA and 107mg DHA.  DHA-rich fish oil, providing 264mg EPA and 1.032 mg DPA.  **Dose:** 4x 500mg per day  **Duration (wk):** 16 weeks | **Description:** Safflower oil, providing 1.467 mg LA  **Dose:** 4x 500mg per day  **Duration (wk):** 16 weeks | ADHD Core symptoms, parent rated, end of treatment  Behavioral difficulties, parent rated, end of treatment  Side effects (gastrointestinal discomfort) | Increasing erythrocyte DHA and EPA via dietary supplementation may improve behavior, attention, and literacy in children with ADHD. |
| Moghaddam 2017 | **Description:** 0.3mg/kg methylphenidate per day was started twice a day and it reached 1mg/kg within two weeks.Treatment with omega-3 capsules containing 180mg EPA and 120mg DHA was started concurrently  **Duration (wk):** 8 weeks | **Description** Methylphendiate + placebo  **Duration (wk):** 8 weeks | ADHD Core symptoms, parent rated, end of treatment  ADHD Core symptoms, teacher rated, end of treatment | This study shows that PUFA is an efficient nutrient to treat ADHD and it can be used to treat  patients. |
| Mohammadzadeh 2019 | **Description:** Methylphenidate and omega-3.  **Dose:** Omega-3 EPA capsules (180 mg) and DHA (120 mg) were given to patients as pockets (1 and 2) as the same gel-shaped capsules.  **Duration (wk):** 8 weeks | **Description:** Methylphenidate and placebo  **Dose:** placebo (olive oil) were given to patients as pockets (1 and 2) as the same gel-shaped capsules.  **Duration (wk):** 8 weeks | ADHD Core symptoms, parent rated, end of treatment  Side effects (Diarrhea, Nausea, gastrointestinal discomfort) | Our results demonstrate that a specific dose of omega-3 for 8 weeks had no effect on ADHD. |
| Perera 2012 | **Description:** The active treatment (commercially marketed as Vegepa) was a capsule containing o3 and o6 (fish oil and cold-pressed evening primrose  oil in the ratio 1.6:1, o3 ¼ 296.37 mg, o6 ¼ 180.75 mg).  All participants continued taking  methylphenidate (immediate-release preparation 0.7-1 mg/kg/d) and  continued the home- and classroom-based behavioral interventions  throughout the study period. (Immediate-release methylphenidate is  the only preparation available in Sri Lanka.) In addition, both groups were administered micronutrients in recommended doses for age as tablets to avoid any confounding effect from deficiency states. Any  other medication the child was already taking was continued. None were introduced to new medications during the study.  Dose: The dose throughout the study was 2 capsules per day in 2 doses, administered by mothers. All participants were supplied with capsules for 30 days at a time. At the end of each 30 days participants were reviewed by the authors, who assessed for any adverse effects, encouraged compliance, and provided another supply of capsules.  **Duration:** 6 months. | **Description:** A capsule of identical appearance containing sunflower oil was used as the placebo.  All participants continued taking  methylphenidate (immediate-release preparation 0.7-1 mg/kg/d) and  continued the home- and classroom-based behavioral interventions  throughout the study period. (Immediate-release methylphenidate is  the only preparation available in Sri Lanka.) In addition, both groups  were administered micronutrients in recommended doses for age as tablets to avoid any confounding effect from deficiency states. Any  other medication the child was already taking was continued. None were introduced to new medications during the study.  **Duration:** 6 months. | ADHD Core symptoms, parent rated, end of treatment | The combination of o3 and o6 was safe and effective in  improving behavior and learning in the group that was studied.  The current study is a pilot, and replication of the findings is  required before we can advocate supplementation as a routine  practice for children with behavior and learning difficulties that are refractory to standard managements. At the same time, it is possible to infer from the results that the participants of the study may have benefitted further if treatment with o3 and  o6 had been continued. |
| Raz 2009 | **Description:** The EFA supplements chosen for this study (produced by  TransCulture, Japan) were in the form of softgel capsules.  Each capsule contained: 240 mg of linoleic acid (LA), 60 mg of  a-linolenic acid (ALA), 95 mg of mineral oil, and 5 mg of  a-tocopherol (as an antioxidant). Each subject was asked to  consume one EFA capsule b.i.d, reaching a daily EFA amount  of 600 mg.  **Duration (wk):** 7 weeks | **Description:** The placebo chosen for this study was vitamin C in the form of tablets (produced by Perrigo Company, MI). Each tablet contained 500 mg of ascorbic acid. Each subject was asked to consume one placebo tablet b.i.d, reaching a daily vitamin C amount of 1,000 mg.  **Duration (wk):** 7 weeks | ADHD Core symptoms, teacher rated, end of treatment  Side effects (Nausea) | Analysis of variance for repeated measures revealed that both treatments ameliorated some of  the symptoms, but no significant differences were found between the groups in any of the treatment effects. |
| Rodriguez 2019 | **Description:** The DHA studied supplement consisted of a banana-fla-vored emulsion (4.7 g, 5 mL sachets) (Brudy NENEmulsión; Brudy Lab, S.L., Barcelona, Spain), registeredas a“food for special medical purposes”at the SpanishAgency of Consumption, Food Security, and Nutrition(AECOSAN), and requiring medical prescription andsupervision of treatment. This supplement has a high-con-tent DHA triglyceride having a high antioxidant activitypatented to prevent cellular oxidative damage.32,33Eachsachet provided a combination ofω-3 fatty acids (DHA1,000 mg, EPA 90 mg, and docosapentaenoic acid 150mg), vitamin E (D-alpha-tocopherol) 4.5 mg as an antiox-idant, and carbohydrates 0.94 g (fructose 0.46 g). Doseswere 1 sachet/day in children weighing≤32 kg and 2sachets/day in those weighing >32 kg.  **Duration:** 6 months | **Description: T**he placebo sachets had the same composition and were indistinguishable from the active product. They were composed of the same amount of olive oil with banana flavor to give a similar taste and smell.  **Duration:** 6 months | ADHD Core symptoms, parent rated, end of treatment | This study provides further evidence of the beneficial effect of supplementation with ω-3 DHA in the management of ADHD. |
| Salehi 2016 | **Description:** daily Ritalin plus one omega-3 capsule (produced by Zahravi Pharmaceutical Company in Iran) based on the instruction of 100 mg eicosapentaenoic fatty acid for children <25 kg, 200 mg for 26–35 kg, and 400 mg for children >35 kg/day, respectively.  **Duration (wk):** 8 weeks | **Description:** daily Ritalin plus placebo.  **Duration (wk):** 8 weeks | ADHD Core symptoms, parent rated, end of treatment | Zinc supplementation accompanied by the main treatment significantly improves symptom of attention deficit disorder subtype of ADHD. However, omega-3 supplementation was superior to zinc and placebo in  the clinical improvement of ADHD. |
| Sinn 2007 | **Description:** The LC-PUFA capsules used for the study were eye qTM, each containing 400 mg fish oil and 100 mg evening primrose oil with active ingredients eicosapentaenoic acid (EPA)  (93 mg), docosahexaenoic acid (DHA) (29 mg), gamma linolenic acid (GLA) (10 mg), and vitamin E (1.8 mg).  Children were required to  take six capsules per day.  The multivitamin/mineral (MVM) supplement were fruit-flavored  chewable tablets. The tablets contained active ingredients:  vitamin A 175 IU, thiamine nitrate 700 g, vitamin B2 1.1  mg; vitamin B6 1.3 mg, nicotinamide 12 mg, vitamin C 60  mg, vitamin D3 100 IU, vitamin B12 1.5 g, vitamin E 6 IU,  biotin 50 g, vitamin B5 2.7 mg, folic acid 100 g, calcium  hydrogen phosphate anhydrous 33.9 mg, ferrous fumarate  7.5 mg, magnesium oxide 8.32 mg, manganese sulfate 77  g, zinc oxide 1.25 mg, copper gluconate 178.6 g, and  potassium iodide 118 g.  **Duration (wk):** 15 weeks | **Description:** Placebo capsules contained palm oil.  Children were required to  take six capsules per day.  **Duration (wk):** 15 weeks | ADHD Core symptoms, parent rated, end of treatment | These results  add to preliminary findings that ADHD-related problems with inattention, hyperactivity, and impulsivity might  respond to treatment with PUFAs and that improvements may continue with supplementation extending to 30  weeks. |
| Stevens 2003 | **Description:** PUFA supplement: 480 mg DHA, 80 mgEPA, 40 mg arachidonic acid (AA), 96 mg GLA, and 24 mg α-tocopheryl acetate.  **Dose:** 720mg  **Duration (wk):** 16 weeks | **Description**: 0.8g olive oil  **Duration (wk):** 16 weeks | ADHD Core symptoms, parent rated, end of treatment  ADHD Core symptoms, teacher rated, end of treatment  Behavioral difficulties, parent rated, end of treatment  Behavioral difficulties, teacher rated, end of treatment | A clear benefit  from PUFA supplementation for all behaviors characteristic  of AD/HD was not observed; however, treatment effects for  conduct (parents) and attention (teachers), together with clinical improvements in Oppositional/defiant behavior, along  with significant associations between the magnitude of the  change in RBC FA and vitamin E vs. improvements for some  behavioral outcome measures support further research into  these relationships. |
| Vaisman 2008 | **Description:** 250mg EPA, DHS esterified to PL n-3 300mg + 4-7mg citrus oil extract + rosemary extract, ascorbyl palmite and natural tocopherols or fishoil  **Dose: 55**0 mg  **Durartion (wk):** 12 weeks | **Description:** rapeseed oil  **Durartion (wk):** 12 weeks | ADHD Core symptoms, parent rated, end of treatment | Consumption of EPADHA esterified to different  carriers had different effects on the incorporation of these FAs in  blood fractions and on the visual sustained attention performance in children. |
| Voigt 2001 | **Description:** an algae-derived triglyceride capsule (DHASCO;  Martek Biosciences Corporation,  Columbia, Md), providing 345 mg of DHA per day  **Duration:** 4 months | **Description:** placebo  Capsule  **Duration:** 4 months | No relevant outcomes | A 4-month period of DHA supplementation (345 mg/d) does not decrease symptoms of ADHD. |
| Widenhorn Muller 2014 | **Description:** OMEGA-3  Dose: 600 MG EPA, 120MG DHA  Durartion (wk): 16 weeks | **Description:** olive oil  **Durartion (wk):** 16 weeks | ADHD Core symptoms, parent rated, end of treatment  ADHD Core symptoms, teacher rated, end of treatment | Supplementation with the omega-3 fatty acid mix increased EPA and DHA concentrations in  erythrocyte membranes and improved working memory function, but had no effect on other cognitive  measures and parent- and teacher-rated behavior in the study population. Improved working memory  correlated significantly with increased EPA, DHA and decreased AA (arachidonic acid). |
